# Supplementary material for: Dietary Intake of the Italian PHIME Infant Cohort: How We Are Getting Diet Wrong from as Early as Infancy
Source: Nutrients. 2021 Dec 10;13(12):4430. doi: 10.3390/nu13124430 (PMC8709248; doi:10.3390/nu13124430)
Supplement: Supplementary file 1 [file nutrients-13-04430-s001.zip › nutrients-1460914-supplementary.pdf]

Table S1. Distribution of macro and micronutrients intake of children divided by sex (n = 389)

	FEMALES (n = 204)		MALES (n = 185)		
	Mean±SD	Median(IQR)	Mean±SD	Median(IQR)	*p*-value[1]
Energy (kcal/day)	892.5±190.1	855.9(274.8)	942.0±199.9	927.1(283.4)	*0.04*
Total proteins (g/day)	38.1±10.0	37.0(12.6)	37.5±10.1	36.9(12.9)	0.72
Total lipids (g/day)	33.1±8.4	32.0(11.4)	34.8±10.4	33.2(14.3)	0.18
Saturated fatty acids (g/day)	13.8±4.0	13.4(5.9)	14.4±5.2	13.6(6.9)	0.53
Monounsaturated fatty acids (g/day)	11.3±3.6	11.1(5.0)	11.9±4.5	11.3(5.3)	0.42
Oleic acid (g/day)	10.0±3.4	9.6(4.8)	10.5±4.3	10.1(4.8)	0.21
Polyunsaturated fatty acids (g/day)	3.1±1.2	3.0(1.3)	3.3±1.4	3.0(1.7)	0.17
Linoleic acid (g/day)	2.3±1.0	2.1(1.0)	2.5±1.2	2.2(1.4)	0.06
Linolenic acid (g/day)	0.4±0.1	0.4(0.2)	0.5±0.2	0.4(0.2)	0.25
Available carbohydrates (g/day)	117.9±30.8	114.3(37.6)	127.8±28.4	124.6(36.6)	*<0.05*
Soluble carbohydrates (g/day)	51.5±17.3	48.4(19.1)	56.3±16.0	55.2(19.7)	*<0.05*
Starch (g/day)	53.0±22.6	50.6(32.5)	59.4±22.5	57.8(28.8)	<0.05
Fibre (g/day)	6.9±2.9	6.5(3.9)	7.8±2.9	7.4(3.8)	*<0.05*
Cholesterol (mg/day)	118.5±41.1	119.0(53.3)	121.0±44.2	117.7(49.4)	0.58
Sodium (mg/day)	858.2±290.3	819.1(397.7)	904.1±331.4	867.1(433.3)	0.20
Potassium (mg/day)	1393.2±396.4	1379.6(479.9)	1444.8±429.2	1392.5(587.4)	0.34
Calcium (mg/day)	649.4±220.5	644.1(290.4)	636.3±250.0	592.3(3)	0.29
Iron (mg/day)	4.5±1.6	4.3(1.9)	4.6±1.5	4.6(1.7)	0.10
Zinc (mg/day)	4.4±1.3	4.2(1.6)	4.4±1.3	4.4(1.9)	0.82
Vitamin B1 (mg/day)	0.6±0.2	0.6(0.2)	0.6±0.2	0.6(0.2)	0.97
Vitamin B2 (mg/day)	1.1±0.3	1.0(0.4)	1.0±0.3	1.0(0.5)	0.35
Vitamin B6 (mg/day)	1.0±0.3	1.0(0.3)	1.0±0.3	1.0(0.4)	0.73
Vitamin B12 (μg/day)	2.4±1.1	2.3(1.3)	2.2±1.1	2.0(1.5)	*0.01*
Vitamin C (mg/day)	55.0±37.9	43.6(38.9)	56.1±31.2	49.0(35.3)	0.22
Vitamin D (μg/day)	1.1±1.5	0.6(0.5)	1.1±2.6	0.6(0.6)	0.24
Vitamin E α-TE (mg/day)	3.3±1.4	3.1(1.8)	3.7±1.9	3.3(2.0)	0.05
Retinol (μg/day)	193.1±78.5	191.7(103.6)	187.0±93.9	175.9(119.5)	0.23
Retinol Eq. (μg/day)	509.2±227.9	469.6(287.6)	515.2±231.0	490.6(301.7)	0.69
Niacin (mg/day)	6.7±2.6	6.4(2.7)	6.4±2.2	6.1(2.7)	0.42
Folate (μg/day)	118.7±43.8	114.1(52.4)	125.0±44.3	119.0(53.6)	0.14

[1]Statistical significant differences between sex were assessed using Wilcoxon-Mann Whitney test; p-value<0.05
Abbreviation: STD, standard Deviation; IQR, InterQuartile Range
